# The London Regional Committee

**Published:** 1920-12-11

**Authors:** Godfrey H. Hamilton

**Affiliations:** Barnes, S.W.


*To the Editor of* The Hospital.

### The London Regional Committee.

*To the Editor of* The Hospital.

Sir,—The action of the executive of the British Hospitals Association in stipulating that members of the new London Regional Committee should be of the governing body of hospitals, thereby excluding salaried officers, is not authorised by the resolution of the meeting of December 2.

I was present throughout the discussion, and except for one observation by one speaker such a qualification was never even mentioned. Had it been part of the scheme I know that there were a number in the hall who would have opposed it. If the action of the executive is persisted in much of the usefulness hoped of the Committee is, to my mind, thrown away.

This is an addition to that tradition of muddled action, that faculty of just missing to be useful, which unfortunately attaches to the British Hospitals Association, chiefly, I believe, for lack of immediate sources of information of inside hospital knowledge, a condition which now unfortunately vitiates the present scheme. It was hoped by several that the Regional Committee might, temporarily at least, take the place of that Central Board advocated by the President of the Hospital Officers' Association in his inaugural address. Apparently hospital men must now look in the direction indicated by him.—Yours faithfully,

Godfrey H. Hamilton.

Barnes, S.W., December 8, 1920.

